# LC-MS/MS-based metabolomics and proteomics reveal the intervention of Kangnian decoction on the postoperative intestinal adhesion of rats

**DOI:** 10.3389/fphar.2024.1382760

**Published:** 2024-09-16

**Authors:** Liang Jin, Yuan Zhao, Xiaojing Qian, Lingyun Pan, Long Chen, Jingwen Feng, Xinhua Liu, Xiaotong Lu

**Affiliations:** ^1^ Xinhua Hospital Affiliated to Shanghai Jiaotong University School of Medicine, Shanghai, China; ^2^ Shanghai University of Traditional Chinese Medicine, Shanghai, China

**Keywords:** postoperative intestinal adhesion, Kangnian decoction, metabolomics, proteomic analysis, MAPK signaling pathway

## Abstract

**Background:**

Postoperative Intestinal Adhesions (PIAs) remain a significant complication of abdominal surgery that can cause pain, infertility, and a potentially lethal bowel obstruction. Kangnian (KN) decoction, a Traditional Chinese Medicine prescription, has been shown to be effective in treating PIAs. Nevertheless, its underlying mechanisms remain unclear.

**Objective:**

This study aims to explore the therapeutic effects of KN decoction in a PIA rat model, as well as its potential mechanisms via metabolomics and proteomics analyses.

**Materials and methods:**

60 rats were randomly assigned to six groups: Normal Control (NC), PIA model, Dexamethasone, KN-Low, KN-Medium, and KN-High. The PIA model was created by abdominal surgery under anesthesia. Pathological damage was evaluated through H&E staining and adhesion grading of affected tissues. The levels of serum cytokines (IL-1β, IL-6, TNF-α, and TGF-1), Connective Tissue Growth Factor (CTGF), and Motilin (MTL) in adherent intestinal tissues were detected using ELISA kits. Untargeted metabolomics was used to investigate potential metabolic pathways of the KN decoction intervention in intestinal adhesions and to screen for differential biomarkers. The label-free quantitative proteomics technique was employed to detect Differentially Expressed Proteins and for biological function and pathway enrichment analyses.

**Results:**

In PIA rats, KN decoction significantly improved the pathological injury associated with intestinal adhesions and effectively regulated the blood inflammation indicators. Furthermore, KN presented a favorable anti-fibrotic and protective effect against abdominal adhesions, effectively modifying gastrointestinal motility disorders in PIA rats. We identified 58 variables as potential biomarkers and discovered seven main pathological pathways that may be associated with PIAs. Proteomics analysis revealed 75 DEPs that were primarily involved in Valine, leucine, and isoleucine degradation, the MAPK signaling pathway, and retrograde endocannabinoid signaling.

**Conclusion:**

This study proved that KN reduces intestinal mucosal injury, downregulates inflammatory factors, and alleviates intestinal adhesions, thus protecting the intestinal barrier function in PIA rats. The combination of proteomics and metabolomics provided a feasible approach for unraveling the therapeutic mechanism of KN decoction in PIAs.

## 1 Introduction

With an incidence of about 93%, Postoperative Intestinal Adhesions (PIAs) are the most common complication post-laparotomy (Sahbaz et al., 2014). Intestinal obstruction is the most common complication of intestinal adhesions. Additionally, intestinal adhesion-induced mechanical intestinal obstruction is the primary factor in the pathogenesis of intestinal obstruction.

Surgical trauma is the main cause of intestinal adhesions, with other causes including intra-abdominal infections, ischemia, foreign body retention, congenital anomalies, and postoperative trauma. Although clinicians have explored multiple PIA aspects and achieved some therapeutic progress, solutions for completely preventing intestinal adhesions are yet to be discovered.

Modern pharmacological research has confirmed the potential of Traditional Chinese Medicine (TCM) in promoting postoperative Gastrointestinal (GI) peristalsis, regulating the balance of intestinal flora, improving intestinal blood flow and perfusion, reducing the intestinal segment’s inflammatory response during surgery, and promoting fibrinolysis, thereby alleviating PIAs. To reduce patient pain, Xinhua Hospital developed the Kangnian (KN) decoction, a protocol prescription that has been widely used in clinical practice for decades to prevent PIA occurrence after pediatric abdominal surgery, based on clinical pediatric surgery experience and basic TCM theory. Notably, the KN decoction has been used in the past 5 years to prevent PIAs in more than 1,200 cases, with remarkable clinical effects and minimal adverse reactions. Nevertheless, the effects of KN decoction in intervening intestinal adhesions and its underlying mechanisms are yet to be explored.

Omics techniques with advanced analysis tools (such as proteomics, metabolomics, genomics, and transcriptomics) have been widely used in medical diagnostics and basic research. Compared to traditional methods, omics techniques are more likely to reveal new therapeutic targets (Gioria et al., 2016; Dos Santos et al., 2016). Metabolomics is mainly used to discover biomarkers and their perturbed pathways. This approach can monitor pathological changes in the body, contribute to early intervention in disease processes, and reveal the underlying therapeutic mechanisms. Over the past decades, Mass Spectrometry (MS)-based proteomics has emerged as a potent technology for high-throughput analysis and identification of proteins (Schulze and Usadel, 2010). Furthermore, quantitative proteomics has been proven to be useful in revealing relative differences in protein abundance in normal and disease samples, offering crucial information on molecular interactions, signaling pathways, and biomarker identification in human disease research (Cifani and Kentsis, 2017). In summary, proteomics is mainly used to identify the entire set of de-regulated dynamic proteins, as well as their interactions in a cell or body fluid, whereas metabolomics is mostly used to analyze the level changes of low molecular weight compounds in cellular and extracellular fluids (Dos Santos et al., 2016; Heijne et al., 2005).

Herein, we employed Ultra-High-performance Liquid Chromatography coupled with a Linear Ion Trap-orbitrap Mass Spectrometry (UPLC-LTQ-Orbitrap-MS/MS)-based metabolomics approach to detect biomarkers in rat tissue and their perturbed pathways, as well as changes following KN decoction intervention. Meanwhile, the Label Free Quantification (LFQ) -based proteomics technique was used to screen for Differentially Expressed Proteins (DEPs) in the bio-samples and observe their variations after KN decoction treatment. Finally, data from both analyses were integrated to elucidate the effects of KN decoction in preventing PIAs and illuminate the mechanisms underlying its intervention in the disease.

## 2 Materials and methods

### 2.1 Description of the botanical drugs in KNF decoction and relevant extraction process

The Kangnian (KN) decoction is an empirical prescription used by Xinhua Hospital for the prevention of intestinal adhesions after surgery, which is composed of Rhei Radix Et Rhizoma (the dried roots and rhizomes of *Rheum officinale* Baill.), Cyperi Rhizoma (the rhizome of *Cyperus rotundus* L.), Cannabis Fructus (the fruits of *Cannabis sativa* L.), Trichosanthis Fructus (the fruits of *Trichosanthes kirilowii* Maxim.), Violae Herba (the original plants of *Viola yedoensis* Makino), Liushenqu (Massa Medicata Fermentata), Taraxaci Herba (the radix and original plants of *Taraxacum mongolicum* Hand. -Mazz.), and Lonicerae Japonicae Caulis (the dried stem and branch of *Lonicera japonica* Thunb.), it has been used in Xinhua Hospital for more than 30 years, with remarkable clinical effects and few side effects.

The main active metabolites of the recipe were identified as cichoric acid, chlorogenic acid, strychnine, qinpietin, aloe emodin, rhein acid, emodin, chrysophanol, emodin methyl ether, etc. Based on the HPLC method, a method for the simultaneous determination of the content of multiple components in KN Decoction was established, which provided an experimental basis for the quality control of the KN Decoction (Supplementary Material).

#### 2.1.1 Preparation of the KN decoction

The KN decoction needs to be extracted with an aqueous solution for optimal efficacy. Rhei Radix Et Rhizoma (12 g), Cyperi Rhizoma (10 g), Cannabis Fructus (10 g), Trichosanthis Fructus (10 g), Violae Herba (15 g), Liushenqu (10 g), Taraxaci Herba (30 g) and Lonicerae Japonicae Caulis (12 g) were allowed to stand and soak in 1:10 (w/v) water for 1 h. The extract was then decocted for 1 h using 1:8 (w/v) purified water and repeated two times. The filtrate was mixed and concentrated under reduced pressure (60°C). Finally, the KN decoction was stored at - 20°C for spare use.

### 2.2 Reagents and chemicals

The Dexamethasone Palmitate (DXP) injection (D21014, 4 mg/1 mL/branch, Mitsubishi Tanabe Pharma Corporation, Japan), benzylpenicillin sodium injection (0.48 g/branch, Shandong Lukang Pharmaceutical Co., Batch No. 43210804), 0.9% sodium chloride injection (500 mL, Sinopharm Chemical Reagent Co., Ltd.) and KN decoction were acquired from Xinhua Hospital’s pharmacy (Batch No. 2303028, Shanghai, China). The HPLC-grade acetonitrile and methanol were purchased from Merck KGaA (Darmstadt, Germany). Ultra-pure water was prepared using the Milli-Q water purification system (Millipore Corporation, MA, United States). Formic acid (chromatographic purity) was purchased from CNW Technology (Germany). The Rat TGF-β1 ELISA Kit (Lot No: EK981-96), Rat IL-6 High Sensitivity ELISA Kit (Lot No: EK306HS-96), Rat IL-1β High Sensitivity ELISA kit (Lot No: EK301BHS-96), Rat TNF-α High Sensitivity ELISA kit (Lot No: EK382HS-96), Rat MTL ELISA Kit (Lot No: G20230421SF), and Rat CTGF ELISA Kit (Lot No: G20230419MA) were purchased from Multi Sciences (Lianke) Biotech Co., Ltd. (Zhejiang, China). The H&E staining kit (Lot No: GP1031) was purchased from Service-bio (China). The Enhanced BCA Protein Assay Kit (Cat No: P0010) and Iodoacetamide (IAA; Lot No: 102387291) were purchased from Sigma-Aldrich (St. Louis, MO, United States). Urea and NH4HCO3 were purchased from Sinopharm Chemical Reagent Co. Ltd. (Shanghai, China).

### 2.3 Animals and treatment

Male Sprague Dawley (SD) rats (230 ± 20 g) were purchased from SLAC Laboratory Animal Co. Ltd. (Shanghai, China) and housed under Specific Pathogen-Free (SPF) conditions with *ad-libitum* access to regular food and water. All animal experiments were ethically approved by the Animal Care and Ethics Committee of the Shanghai University of Traditional Chinese Medicine (Approval number: PZSHUTCM210312010).

Herein, 60 rats were equally divided into six groups (n = 10). The experimental groups’ animals (50 rats) were fasted for 12 h. Following that, their abdomens were shaved and sterilized after being anesthetized with isoflurane. The surgery was performed under aseptic conditions. After dissecting and opening the abdomen, the cecum was pulled outward and exposed to the air for 5 min. It was then rubbed with sterile gauze until the plasma membrane ruptured and tiny hemorrhages formed. The cecum was incorporated back into the abdominal cavity when the damaged wound (approximately 2.5 cm × 2.5 cm) was formed. A surgical blade was used to scrape the corresponding abdominal wall until injury was induced. Finally, the abdomen was gradually closed, sutured, and sterilized. Except for the Normal Control (NC) group animals, all rats were intramuscularly treated with 80,000 units of penicillin/250 g of body weight per day. The NC and Model groups received equal amounts of saline through gavage (10 mL/kg). The KN groups (low, medium, and high) received different doses of KN decoction (4.9, 9.8, and 19.6 g/kg, respectively) and the Dexamethasone group was treated with Dexamethasone (10 mg/kg) through abdominal injection once daily for 10 days after the PIA model was constructed. After the last treatment, all rats were fasted for 12 h and then anesthetized with isoflurane to obtain blood samples from the abdominal aorta. The animals were then euthanized. Subsequently, the serum was obtained and centrifuged at 3,800 rpm for 15 min at 4°C. Furthermore, adherent intestinal tissues and the ileum and cecum tissue portions were surgically harvested and stored in a −80°C refrigerator, awaiting further experiments. Special care was taken to minimize animal suffering.

### 2.4 Adhesion grading

The modified Evans adhesion scoring system was used in this study (Sahbaz et al., 2014). This system has five scores (0–4); where grade 0 = no adhesions, grade 1 = one band of adhesions between viscera or between viscera and the abdominal wall, grade 2 = two bands of adhesions between viscera or between viscera and the abdominal wall, grade 3 = more than three bands of adhesions without a direct adhesion of viscera to the abdominal wall, and grade 4 = direct adhesion of viscera to the abdominal wall. The same researcher determined all adhesion scores. Each animal’s adhesion grade was defined as its strongest adhesion per the scoring system.

### 2.5 Histopathological examination

The cecal area and associated adhesive lesions were excised and fixed for 24 h in 4% paraformaldehyde for preservation. Following a routine histological examination process, tissue sections (5 μm thick) were prepared and embedded in the Hematoxylin-Eosin (H&E) staining solution per the manufacturer’s instructions (Service-bio, China). Histopathological changes of inflammatory infiltration and fibrosis in histological sections were observed under a light microscope (Eclipse Ci-L, Nikon, Japan).

### 2.6 Detection of serum IL-1β, IL-6, and TNF-α levels

The frozen serum was first thawed on ice, and then the IL-1β, IL-6, and TNF-α levels were determined separately using the corresponding ELISA kits per the manufacturer’s instructions.

### 2.7 Detection of TGF-β1 and CTGF levels in intestinal adhesion tissues and MTL levels in ileal tissues

Following blood collection from the animals, 0.5 g of adherent intestinal wall tissue was cut on ice, immediately transferred and frozen in liquid nitrogen, and then preserved at −80°C. Following that, a 10% homogenate was prepared by adding saline to a certain mass of adherent tissue. The resulting homogenate was first centrifuged at high speed for 10 min at 4°C, and the supernatant was collected after the second centrifuge at the same temperature and speed for 20 min. The TGF-β1 and CTGF protein content in the tissues was then determined separately per the operational requirements of the ELISA kits. Following the same pre-treatment method, the MTL level was determined using 0.5 g of ileum tissue obtained 2 cm from the ileocecal portion, per the operational requirements of the corresponding ELISA kit.

### 2.8 Protein extraction and digestion

Caecum proteins were extracted and digested with a tissue lysis buffer [7 mM Urea, 2% SDS, 50 mM Tris-HCL, and protease inhibitor cocktail (1x)] (1 mg: 6 μL), using a freezing tissue homogenizer. The samples were then centrifuged at 13,000 rpm at 4°C for 20 min to remove insoluble precipitates. Protein concentration was initially quantified through a BCA assay. Following that, the protein solutions were subjected to overnight acetone precipitation and redissolved in the solution in preparation for the second determination of BCA concentration. The protein solution (100 µg) was treated with DTT (1,4-Dithiothreitol, 20 mM) and reduced for 1 h at 57°C and subsequently with IAA (Iodoacetimide, 1M) for 40 min at 25°C for reductive alkylation. The sample was then added to a 10-kDa filter (VIVACON 500, Sartorius, PN: vn01h02) and washed with 25 mM NH_4_HCO_3_ five times. Following that, trypsin was added to a final substrate-to-enzyme ratio of 30:1 (w/w). The trypsin-digested sample was then incubated at 37°C for 10 h. Finally, the peptide samples were desalted using a Monospin tip before MS analysis.

### 2.9 Nanoflow liquid chromatography-tandem mass spectrometry analysis of proteomics

We performed proteomics analysis on an Accept PepMap RSLC C18 column (75 μm × 25 cm, 2 μm, Nano Viper, 100 A) using a nanoflow HPLC Easy-NLC 1000 system (Thermo Fisher Science). The mobile phases comprised water-0.1% formic acid (A) and acetonitrile-0.1% formic acid (B). The gradients were configured as follows: 0–2 min, 2–8% B; 2–112 min, 8–28% B; 112–114 min, 28–90% B; and 114–120 min, 90% B. Proteome analysis was performed using the Orbitrap Fusion Lumos mass spectrometer (Thermo Fisher Science). The spray voltage was set at 2100 V. The MS observation was obtained at 120,000 resolution and collected from the mass analyzer using the full ion scan mode over a 350–1,600 m/z range. Precursor ions with a High Collisional Dissociation (HCD) degree were selected for fragmentation, with a normalized collision energy of 30%. The separation window was set at 0.7 m/z. The original LC-MS detection data were processed and analyzed with Protein Discoverer 2.4 SP1 software (Thermo Fisher Science) for qualitative and quantitative protein identification, followed by comparison with Nonredundant Protein (NCBI) and UniProt databases (https://www.uniprot.org/).

### 2.10 Bioinformatics analysis

After obtaining quantitative data, the differential proteins (FC > 1.2 or <0.83, *p* < 0.05) were analyzed to identify the associated biological pathways and network interactions. Protein classification, molecular functions, cellular components, and biological pathways were analyzed using the Gene Ontology (http://geneontology.org/) and UniProt (https://www.uniprot.org/) databases. On the other hand, the KEGG (www.genome.jp/kegg/) database and Gene Set Enrichment Analysis (GSEA) were used to enrich the biological functions and identify significant and coordinated changes in DEPs, respectively.

### 2.11 Sample preparation for MS metabolomics analysis

Following thawing of the samples, 50 ± 5 mg of adherent intestinal wall tissue was meticulously weighed and transferred into a centrifuge tube. Subsequently, 1 mL of a pre-cooled mixture comprising acetonitrile/methanol/water (2:5:2) was added, followed by the introduction of 10 μL of an internal standard solution 2-chlorophenylalanine, 300 μg/mL. The mixture was then homogenized with steel beads, and subjected to centrifugation at 12,000 rpm at 4°C for 15 min. Carefully, 200 μL of the supernatant was extracted and transferred for subsequent analysis.

### 2.12 Untargeted metabolomics assay

The separation was performed using Ultra High-Performance Liquid Chromatography (UPLC) coupled with High-Resolution Mass Spectrometry (HRMS; LTQ-Orbitrap Elite, Thermo Fisher Scientific, Germany) and a Waters ACQUITY UPLC HSS T3 column (2.1 mm × 100 mm, 1.8 μm). Solvent A (water-0.1% formic acid) and solvent B (acenotrile-0.1% formic acid) were used as the mobile phases. The elution gradient was configured as follows: 0–1 min, 95% A; 1–2 min, 95%–60% A; 2–7 min, 60%–20% A; 7–11 min, 20%–5% A; and 11–15 min, 5% A. The column temperature, injection volume, and volume flow rate were 40°C, 4 μL, and 0.3 mL/min, respectively. Detection was performed in both the positive and negative ionization modes using an Electrospray Ionization Source (ESI) (Thermo Fisher Scientific, Dreieich, Germany) with spray voltages of 3.8 kV and 4.0 kV, respectively. The full mass scan range was 50–1,000 m/z. The ion source, heater and capillary temperatures were 100°C, 300°C, and 350°C, respectively. High-purity nitrogen was used as both the sheath gas and auxiliary gas at volumetric flow rates of 45 and 15 arb, respectively.

### 2.13 Metabolomics data processing

For peak matching and aligning, the acquired data were processed using the Compound Discoverer 3.30 software (Thermo Fisher Scientific, Dreieich, Germany). The data were then normalized and subjected to Principal Component Analysis (PCA) and Orthogonal Partial Least Squares-Discriminant Analysis (OPLS-DA) using the SIMCA 14.1 system. The criteria for screening the contributing differential metabolites were VIP>1 and *p* < 0.05 of the independent samples t-test. The HMDB 4.0 metabolomics database was used to fingerprint the potential biomarker identification, and metabolic pathway analysis was performed using the MetaboAnalyst (https://www.metaboanalyst.ca) 5.0 software and the KEGG online database.

### 2.14 Statistical analysis

All statistical analyses were performed using SPSS software 20.0. All results were expressed as Mean ± Standard Deviation (SD). One-way ANOVA followed by Tukey’s test was used for multiple comparisons, and results with *p* < 0.05 and *p* < 0.01 were considered statistically significant and highly statistically significant, respectively. Statistical graphs were created with GraphPad Prism 8 (GraphPad Software, CA).

## 3 Results

### 3.1 The KN decoction alleviates PIAs in rats

**FIGURE 1:**
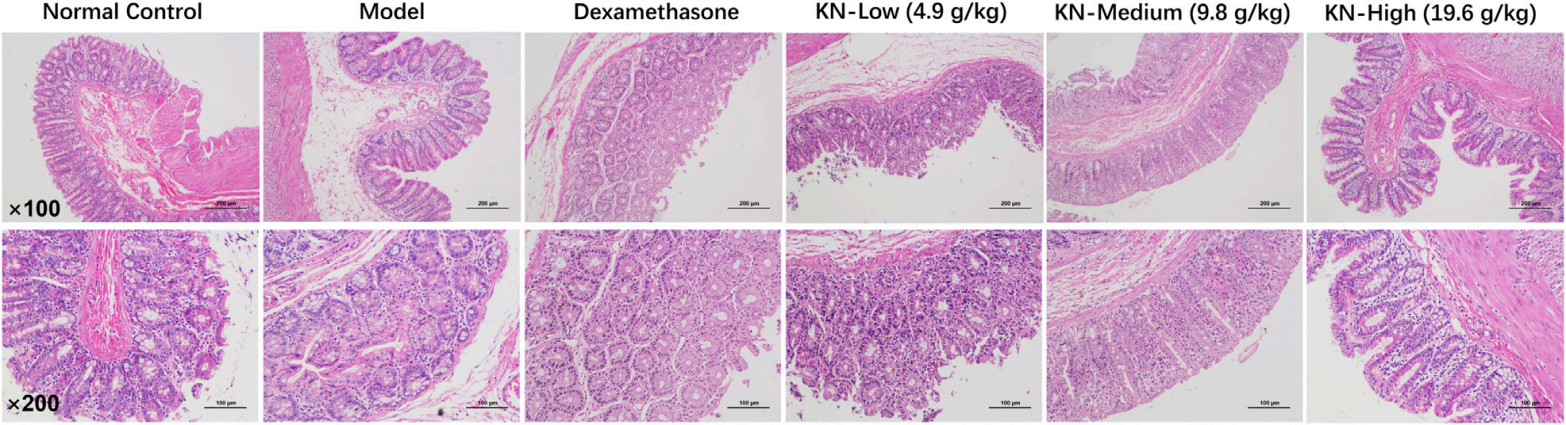
H&E staining results of rat adherent intestinal tissues (×100, × 200).

Table 1 shows the groups’ adhesion scores. Based on the H&E staining results (Figure 1), the NC group had a normal cecum wall structure, with four layers comprising the mucosa, submucosa, muscle, and a plasma layer sequentially arranged from the inside to the outside. In the PIA model group, the damaged intestinal wall generated adherent tissues on the outside, with apparent connective tissue proliferation, severe destruction of the plasma membrane layer, and significant inflammatory cell infiltration. However, rats in the drug administration group exhibited a more apparent cecum wall structure, with minimal loose connective tissues in the submucosal layer, a thinner plasma membrane layer, less fibrous tissue hyperplasia externally, and a lower inflammatory cell infiltration degree compared to the model group. Compared to the DXM group, higher KN decoction concentrations led to a more significant improvement in the animals’ condition, with minimal fibrous tissue proliferation and fewer inflammatory cells, implying that KN decoction effectively attenuated the adhesion in PIA rats.

**TABLE 1:** Adhesion scores in each group ( $\bar{x}$ ± s, n = 10).

Group	Adhesion score (mean ± SD)	*p*-value
Normal Control	0	—
Model	3.40 ± 0.52	0.000000
Dexamethasone	1.20 ± 1.39	0.000192
KN-Low (4.9 g/kg)	2.30 ± 1.06	0.008537
KN-Medium (9.8 g/kg)	1.80 ± 1.03	0.000360
KN-High (19.6 g/kg)	1.50 ± 1.27	0.000357

### 3.2 Determination of the serum IL-1β, IL-6, and TNF-α levels

**FIGURE 2:**
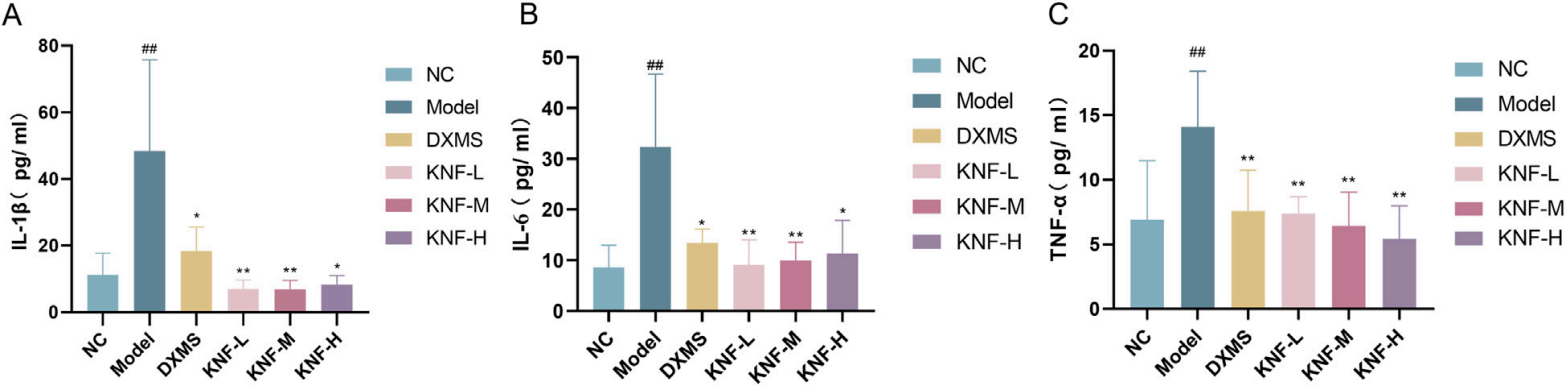
KN decoction alleviates serum inflammatory indicators in PIA rats (n = 10). **(A)** Serum IL-1β levels measured in rats; **(B)** Serum IL-6 levels measured in rats; **(C)** Serum TNF-α levels measured in rats. ^##^ *p* < 0.01 compared to the Normal Control. ^∗^ *p* < 0.05, ^∗∗^ *p* < 0.01 compared to the Model. NC: normal control; Model: PIAs model; DXMS: Dexamethasone; KNF-L: low dose of KN Decoction (4.9 g/kg); KNF-M: medium dose of KN Decoction (9.8 g/kg); KNF-H: high dose of KN Decoction (19.6 g/kg).

Compared to the NC group, the model group exhibited significantly higher IL-1β, IL-6, and TNF-α levels (Figure 2). However, during treatment, the DXM and KN-treated groups exhibited significantly lower IL-1β, IL-6, and TNF-α levels than the model group (*p* < 0.05, *p* < 0.01). These findings demonstrated that KN decoction effectively downregulated blood inflammation indicators in rats with intestinal adhesions. Notably, this anti-inflammatory effect was superior to that of DXM.

### 3.3 Determination of TGF-β1, CTGF, and MTL levels

**FIGURE 3:**
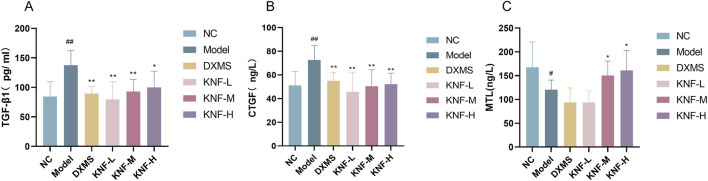
Effect of KN decoction on tissue fibrosis and intestinal protective effects in PIA rats (n = 10). **(A)** TGF-β1 levels measured in adherent intestinal tissues of rats; **(B)** CTGF levels measured in adherent intestinal tissues of rats; **(C)** MTL levels measured in the ileum of rats. ^#^ *p* < 0.05, ^##^ *p* < 0.01 compared to the Normal Control. ^∗^ *p* < 0.05, ^∗∗^ *p* < 0.01 compared to the Model.

Following the manufacturer’s instructions, ELISA kits were used to separately determine TGF-β1 and CTGF levels in adherent intestinal tissues, and MTL levels in the ileum of rats in each group. Tissue TGF-β1 and CTGF levels were significantly higher in the model group than in the NC group (Figures 3A, B), implying that the PIA model was successfully established. Certain peritoneal injuries affected fibrinolytic activity and caused fibrosis, leading to the increased production of cytokines and growth factors, including TGF-β1 and Connective Tissue Growth Factor (CTFG), but the KN decoction intervention gradually reversed this trend. Moreover, the low-concentration KN decoction (4.9 g/kg) had already presented a noticeable anti-fibrotic effect and apparent protection against abdominal adhesions.

Compared to the NC group, the ileal tissues of the model group rats exhibited a significantly lower MTL content (*p* < 0.05). All 3 KN decoction dosages (4.9, 9.8, and 19.6 g/kg) enhanced the intestinal propelling function (*p* < 0.05), indicating that KN decoction could effectively modify GI motility disorders in PIA rats (Figure 3C).

### 3.4 Multivariate statistical analysis

**FIGURE 4:**
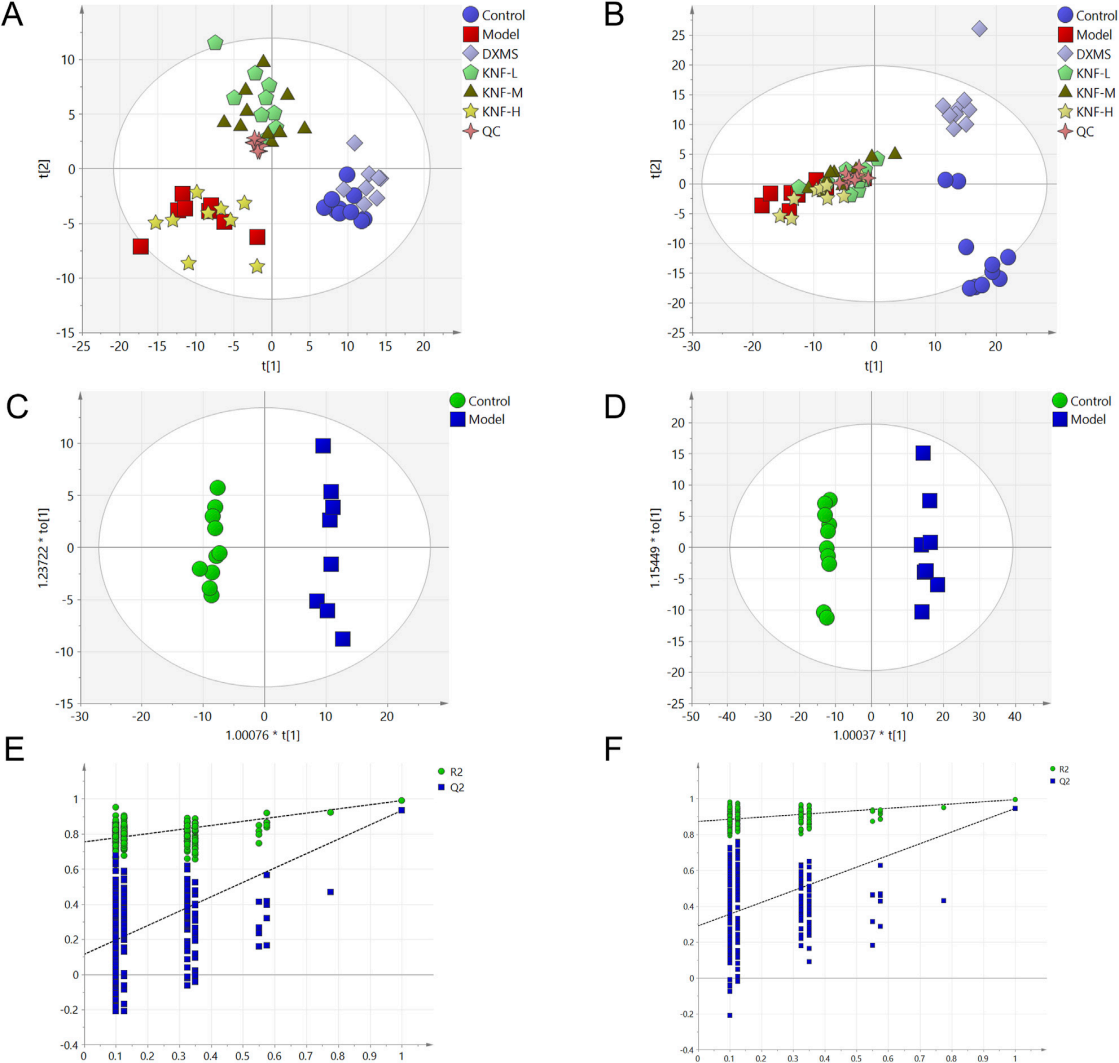
Multidimensional statistical analysis of tissue metabolites. **(A)** PCA score plot in negative ion mode; **(B)** PCA score plot in positive ion mode; **(C)** OPLS-DA score plots from the NC group and model group (n = 10) in negative ion mode; **(D)** OPLS-DA score plots from the NC group (n = 10) and model group (n = 10) in positive ion mode; **(E)** the permutation tests in negative ion mode (n = 200); **(F)** the permutation tests in positive ion mode (n = 200).

Herein, two well-established supervised multivariate statistical analysis methods, PCA and OPLS-DA, were used to obtain information for sorting and identifying metabolites. The PCA score plots (Figures 4A, B) depict each group’s two-dimensional distribution in positive and negative ion modes. The figures show clustered QC samples, indicating that the instrument was stable and the data were reliable. The remaining six groups also demonstrated distinct clustering. The distinct separation between the NC and model groups suggested that PIA rats experienced significant tissue metabolic disturbance. Furthermore, the four treatment groups tended to revert to the blank group’s state, implying that KN decoction effectively improved the model group’s deviation. The R2X values in the positive and negative ion modes were 0.563 and 0.619, respectively. The value was greater than 0.5, indicating that the model was more effective and with a better explanation rate. Inter-group variations in endogenous metabolites were further investigated using OPLS-DA (Figures 4C, D). The model correlation coefficient R2Y values were 0.994 (positive) and 0.998 (negative), and the Q2 values were 0.938 (positive) and 0.956 (negative). The permutation test revealed no over-fitting (Figures 4E, F). This finding confirms the model’s high degree of prediction and accuracy. Differential metabolites were screened using the VIP>1, Fold Change>1.2 or <0.83, and *p* < 0.05 criteria, yielding 58 significantly altered differential metabolites (Supplementary Table S1).

### 3.5 Metabolomic profiling and pathway analysis revealed the metabolic signature of the KN decoction-intervened PIAs

**FIGURE 5:**
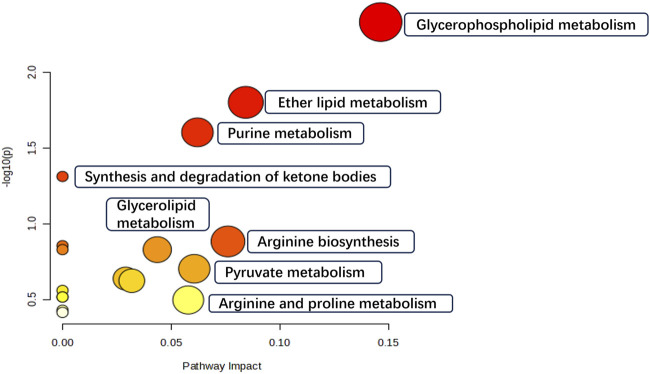
Metabolic differentials pathway enrichment analysis.

We finally identified 58 variables as potential biomarkers. Compared to the NC group, 29 of the identified biomarkers were highly expressed, while the rest had low expression levels in the model group. The MetaboAnalyst 5.0 platform was used to identify the related pathways. The enrichment results revealed 17 target pathways with marked perturbations. Among them, seven main pathological processes (glycerophospholipid metabolism, ether lipid metabolism, purine metabolism, synthesis and degradation of ketone bodies, arginine biosynthesis, glycerolipid metabolism, and pyruvate metabolism) were involved in intestinal adhesion development, indicating the role of multiple pathway dysfunctions in the pathological process of PIAs (Figure 5).

However, after the KN decoction treatment, the differential metabolite content was significantly regulated, and the metabolic pathway disorders were ameliorated, implying that KN decoction exerted a significant regulatory and mitigating effect on PIA tissue metabolism disorders in rats.

### 3.6 Proteomic analysis and identification of DEPs

**FIGURE 6:**
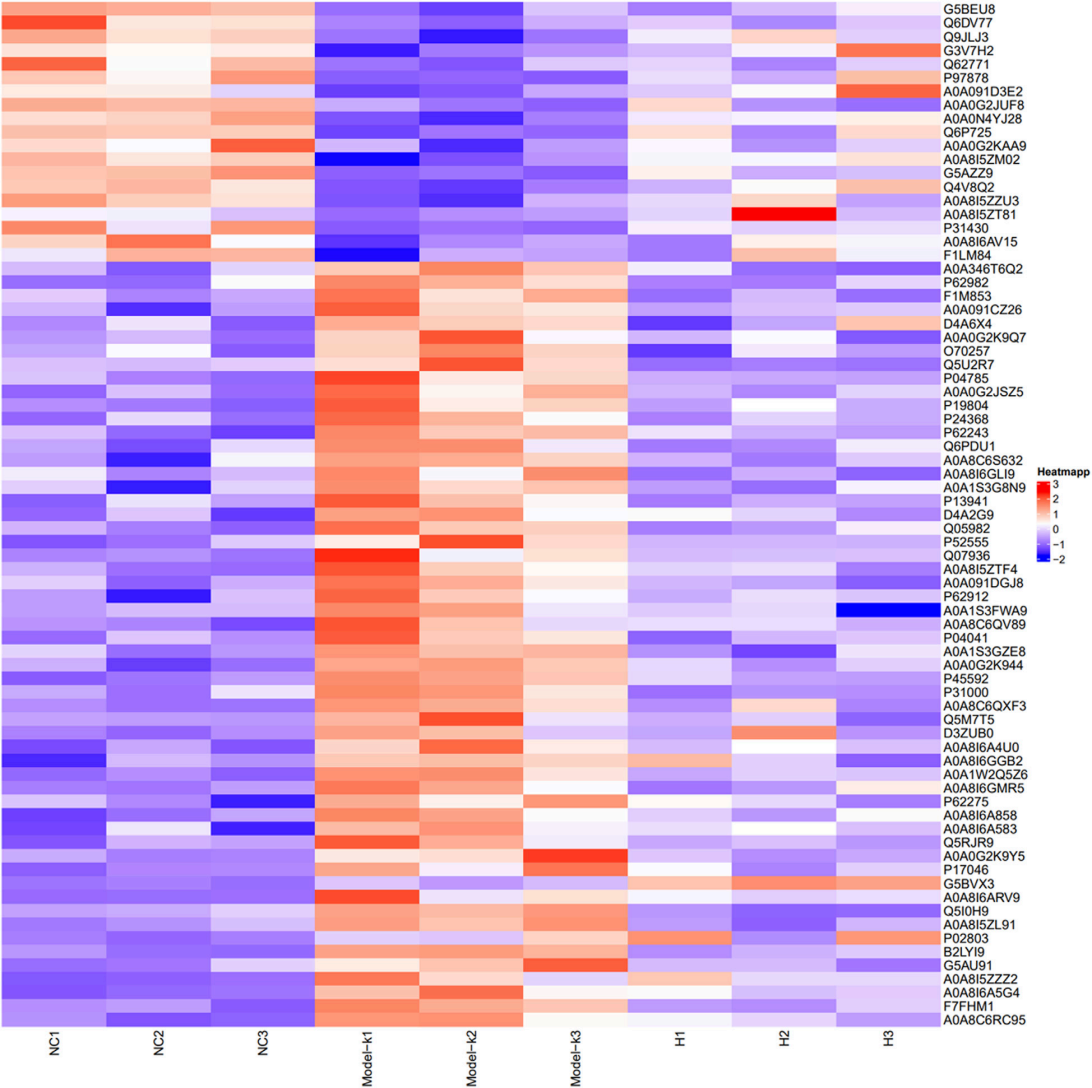
Heat map presenting DEPs between groups (Red represents upregulated, and blue represents downregulated in protein expression).

Using LFQ technology, quantitative proteomics was employed to examine the proteome differences in intestinal adhesion tissues across different groups. The Fold Change >1.2 or <0.83 and *p* < 0.05 criteria were used to screen differential proteins. The relative protein expression values were compared across the groups to identify the DEPs that were of great interest with regard to their possible role in disease progression, as well as their potential capacity in disease diagnosis and revelation of molecular therapeutic targets. Herein, 5,180 proteins were identified. As shown by the heat map (Figure 6), the same proteins exhibited consistent expression trends within groups, with clear differences in expression between groups. Supplementary Table S2 shows the quantified proteins. Among the quantifiable proteins, 75 exhibited a significant difference between the model group and both the NC and KN decoction groups. In the PIA model, 55 and 20 proteins were upregulated and downregulated, respectively. However, after the KN decoction treatment, the protein expression trend was significantly modulated, indicating that KN decoction exerted a regulatory effect on the signaling pathways of PIA rats.

### 3.7 Bioinformatics analysis

**FIGURE 7:**
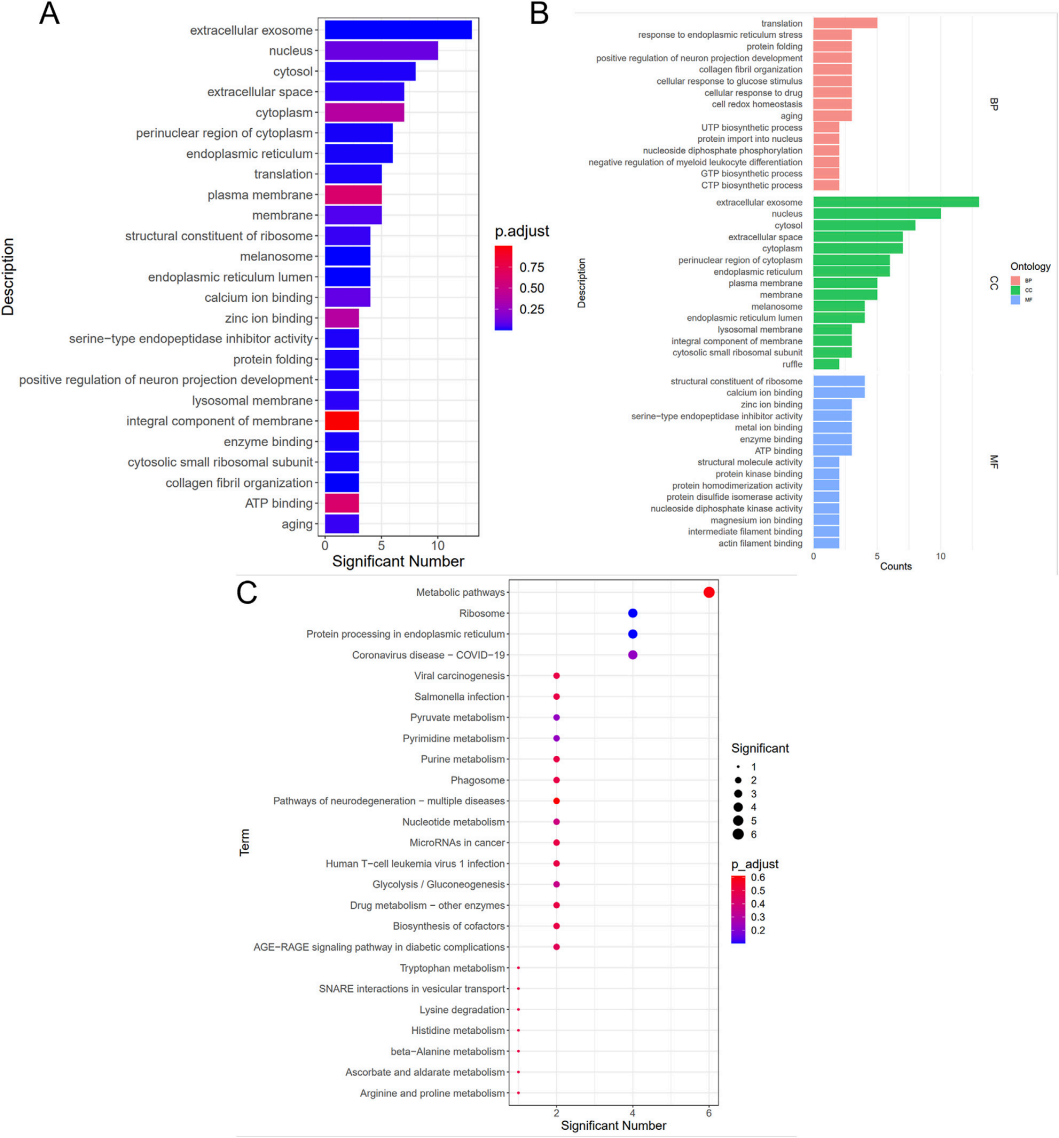
Bioinformatics analysis of GO **(A)**, **(B)**, and KEGG** (C)**.

**FIGURE 8:**
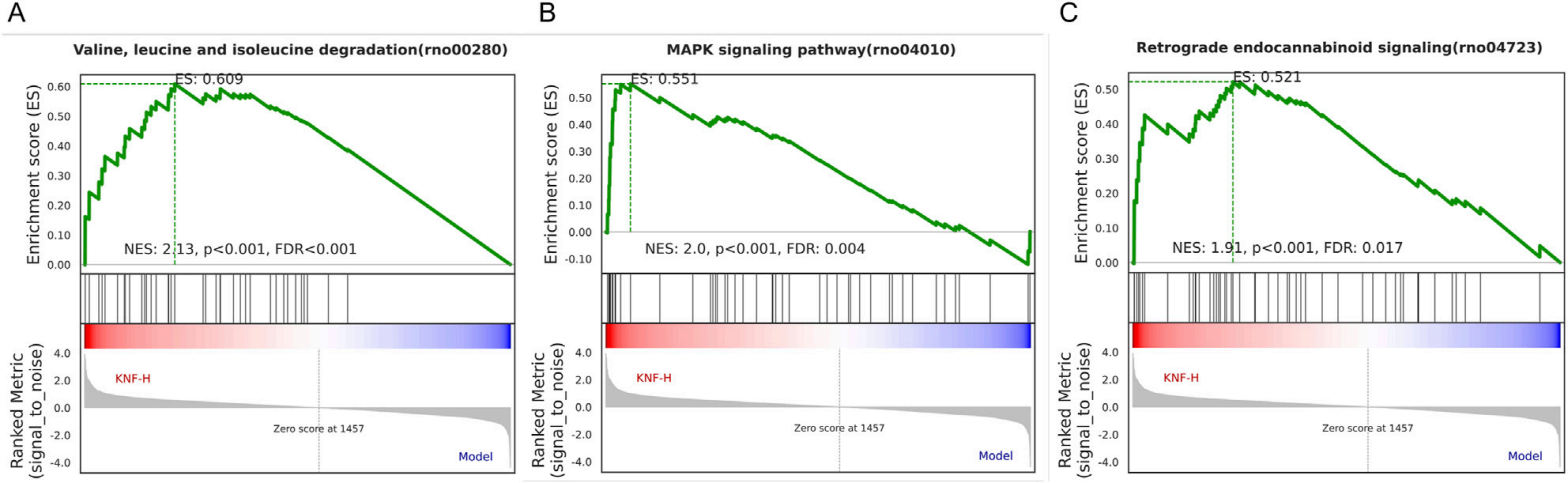
GSEA analysis of Valine, leucine, and isoleucine degradation **(A)**, MAPK signaling pathway **(B)**, and Retrograde endocannabinoid signaling **(C)**.

As presented in Figure 7, GO (7A, 7B) and KEGG (7C) enrichment analyses were performed. In Figures 7A, B, smaller *p*-values and higher blue saturation represent significant differences in biological function, with longer bars representing a higher number of enriched proteins for the specific biological function. The differential proteins were primarily involved in translation, Endoplasmic Reticulum Stress (ERS) response, protein folding, positive regulation of neuron projection development, collagen fibril organization, and cellular response to glucose stimulus, among other biological processes. On the other hand, the DEPs were concentrated in cellular components such as extracellular exosome, nucleus, cytosol, cellular space, cytoplasm, perinuclear region of the cytoplasm, and so on. Regarding molecular function, the differential proteins were mainly concentrated in the structural ribosome constituents, calcium/zinc ion binding, serine-type endopeptidase inhibitor activity, and enzyme binding. The KEGG pathway analysis results are shown in Figure 7C, with the vertical coordinates indicating the pathways enriched for DEPs. The bubble diagram was constructed using *p*-values and the number of differential proteins enriched to each pathway. According to the findings, the proteins were mainly involved in metabolic pathways, ribosomes, protein processing in the endoplasmic reticulum, viral carcinogenesis, and other pathways. Specific biologically significant gene sets that are markedly associated with each of the two sample sets in terms of expression or other measures could be identified through GSEA. In contrast, GO analysis can only be used to identify significant differential genes, as it screens for differential genes and then determines the annotated pathways in which the differential genes are enriched. On the other hand, GSEA is designed to identify genes with synergistic differences in the expression matrix of all genes, and from the gene set enrichment perspective, GSEA allows for the comprehensive assessment of the effects of subtle yet coordinated changes on biological pathways. The enrichment results associated the genomes revealed herein with valine, leucine, and isoleucine degradation, the MAPK signaling pathway, and retrograde endocannabinoid signaling (Figure 8). Furthermore, we used the PathCard platform to individually enrich the pathway information of the screened DEPs, and found that the differential proteins were associated with the MAPK/ERK signaling pathway, the VEGF pathway, EphB-EphrinB signaling, PI3K/AKT signaling, Reactive Oxygen Species (ROS) detoxification, and so on. Briefly, the interruption of these pathways may provide an avenue for developing molecularly targeted PIA therapies.

## 4 Discussion

Adhesion formation, the most common consequence of peritoneal surgery, may increase the risk of secondary surgery and medical burden on patients and society. Adhesions cannot be effectively prevented, and new, larger, and more severe adhesions may occur following surgical treatment. Non-surgical therapies, including pharmacologic interventions, are currently the standard treatment approach for preventing PIAs. In this regard, it is noteworthy that the evidence supporting the use of Chinese medicine to attenuate PIAs should be verified.

Using modern technological means to identify critical proteins and metabolites, as well as the involved pathways that are affected by or respond to a drug in the body is useful in elucidating the interventional mechanisms associated with the specific drugs or their key ingredients. Based on previous clinical practice, this study established a mature and reliable PIA rat model and verified the pharmacodynamic effect of KN decoction in PIA treatment. We also examined the interventional mechanisms of KN decoction by exploring the alterations of protein and metabolic profiles in KN-treated rats using an integrated proteomics and metabolomics approach.

Numerous studies have demonstrated the effectiveness of Chinese medicine therapies in preventing PIA by regulating intestinal adhesion and absorption, as well as inflammation via acting on inflammatory cytokines associated with Inflammatory Bowel Diseases (IBDs) (Li et al., 2022; Becker et al., 2015)**.** Xiao et al. proved that TCM interventions alleviated a gastrointestinal condition by reducing gastric emptying, thereby protecting intestinal barrier function. The treatments also enhanced intestinal mucosal immunity, inhibiting the adhesion of bacteria, viruses, and other pathogens (Xiao et al., 2023). *Rheum palmatum*, the principal medicine of the KN decoction, was used in traditional medicine and continues to be relevant in contemporary herbal therapies. *Rhubarb* extracts have also been extensively studied in the context of treating multiple diseases, including infections, gastrointestinal disorders (Gao et al., 2021), and cancer. Furthermore, *in vivo* experiments have confirmed the anti-inflammatory and antioxidant effects of extracts from *Rheum rhaponticum* and *Rheum rhabarbarum* in human blood plasma and cells (Liudvytska et al., 2023). Moreover, modern pharmacological studies have shown that *Cyperi Rhizoma* exerts various pharmacological effects through its antidepressant, antioxidant, anti-inflammatory, and analgesic properties (Wang et al., 2022).

Under normal physiological conditions, the intestinal mucosa is in a state of ‘controlled’ inflammation maintained by a delicate balance of pro-inflammatory (tumor necrosis factor-α [TNF-α], interferon [IFN]-γ, interleukin [IL]-1, IL-6, IL-1β, IL-12) and anti-inflammatory cytokines (IL-4, IL-10, IL-11). These inflammatory cytokines exist in neuromuscular layers during intestinal inflammation. The mucosal immune system contributes to the occurrence of intestinal inflammation and injury, with cytokines playing a central role in this process (Ardizzone and Bianchi, 2005) and directly affecting intestinal smooth muscle function (Hogaboam et al., 1997). Cytokines may, therefore, be a logical target for PIA therapy using specific cytokine inhibitors. Further analysis was performed to determine whether KN Decoction could reverse the abnormally elevated serum levels of IL-1β, IL-6, and TNF-α. We found that the serum concentration of IL-1β, IL-6, and TNF-α was decreased following KN treatment, indicating that Kangnian Decoction may inhibit intestinal inflammation, restore intestinal smooth muscle function, and alleviate intestinal adhesion damage.

TGF-β1 is a growth factor with a variety of tissue-specific effects (Massague, 1990). It has been shown to stimulate fibroblast proliferation and the production of ECM, indicating that it may be a causative factor of tissue damage (Takehara, 2003; Leask and Abraham, 2004). Experimental data from peritoneal adhesions in both human and animal models of surgically induced adhesions suggest that over-expression of TGF-β may contribute to the development of adhesions (Lower et al., 2000; Emre et al., 2009; Williams et al., 1992). The production of cytokines and growth factors, including connective tissue growth factor (CTGF) leads to the occurrence of fibrotic response (Karaca et al., 2013). The present results demonstrate that KN Decoction treatment decreases the production of TGF-β1, CTGF, and other inflammatory mediators in adherent intestinal tissues, inhibits the migration of cells, and reduces the degree of inflammatory cell infiltration in damaged tissues. Ultimately, it prevents the conversion of collagen fibrinogen into collagen fibrin and inhibits the proliferation of fibrous tissue, thereby reducing the degree of postoperative intestinal adhesions.

**FIGURE 9:**
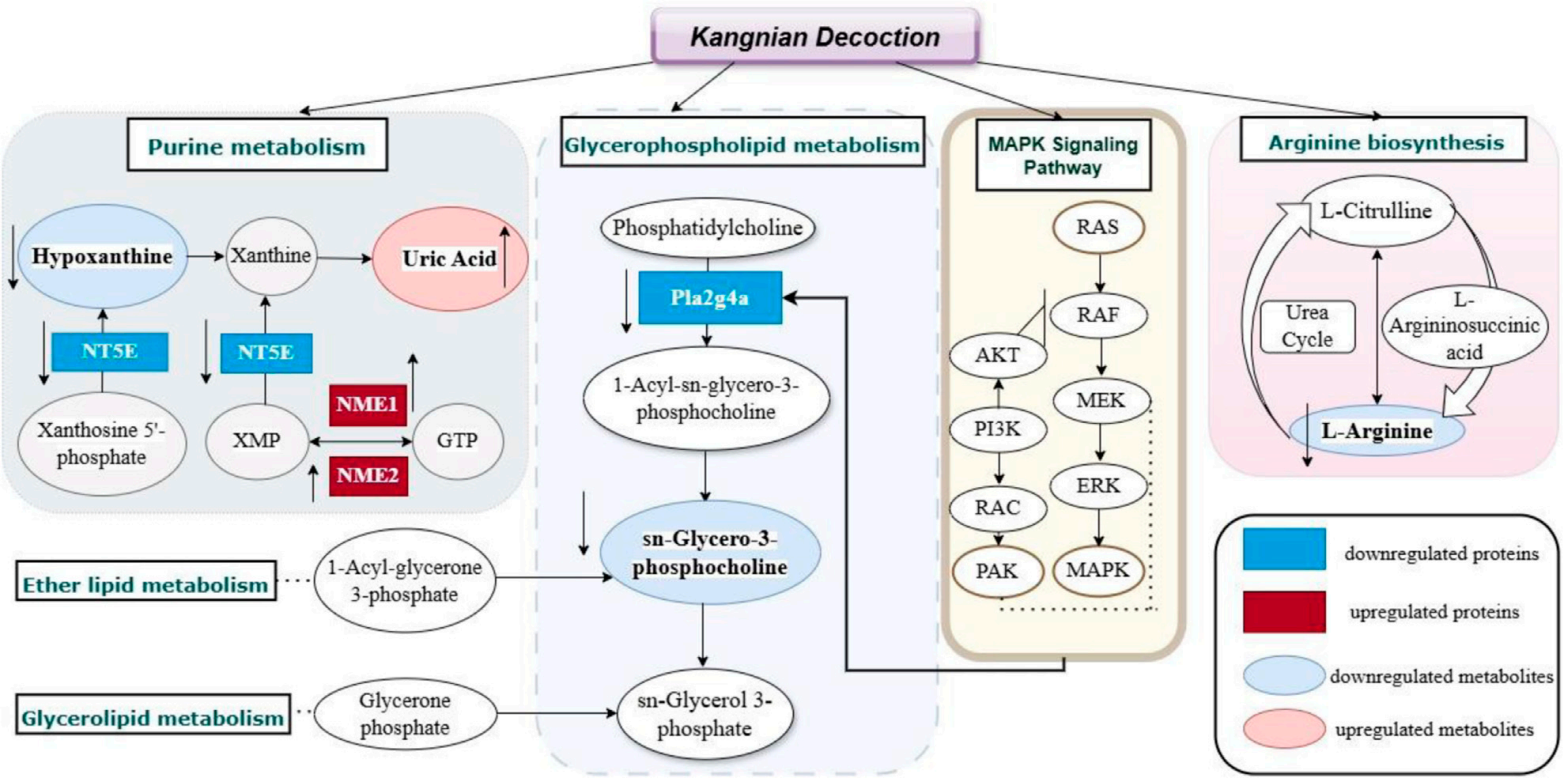
Metabolic-Protein regulatory network diagram of KN Decoction.

In this study, we identified 58 metabolites as potential biomarkers and observed differential expression in 75 proteins. The mechanisms of action of KNF in the treatment of PIAs were further elucidated and mapped through the integration of proteomic and metabolomic datasets (Figure 9). The upregulated proteins and metabolites in the model group were highlighted with red boxes and light red ovals, respectively, while downregulated proteins and metabolites were indicated by blue boxes and light blue ovals, thus providing evidence for analyzing the relationship between proteins and metabolites.

PLA2G4A, which is recruited to perinuclear membranes by Ca2^+^ and activated by extracellular stimuli via the MAPK pathway, specifically cleaves lipids with arachidonic acid (Nicolas et al., 2012). MAPK signaling pathway, crucial for regulating various cellular processes such as inflammation, cell stress response, differentiation, proliferation, metabolism, and apoptosis, was highlighted (Park and Baek, 2022). Research has shown that the RAS protein participates in a variety of cellular signaling pathways, and when it binds to GTP, it increases the phosphorylation activity and activates the downstream proteins. Among such proteins, the RAS/MAPK signaling plays a crucial role (Ratner and Miller, 2015). Our study found that the expression of RAS/MAPK proteins in adhesion tissues of rats in the model group were increased, however, the KN Decoction reversed this change. A previous study performed by McDaniel et al. (McDaniel et al., 2008) identified the key proteins of PAK in cell growth and cytoskeletal dynamics. PAK, which acts as a downstream mediator of RAC1/2, has been recognized as a positive regulator of the MAPK pathway. It regulates the MAPK signaling network and promotes hyperfunctions, particularly in active RAS-dependent proliferation via the PAK1/Erk pathway (Khare et al., 2013). Consequently, we postulated that the strategy of modulating the MAPK signaling pathway to activate downstream metabolites and influence associated lipid metabolism and inflammatory factors could serve as an effective approach to prevent the onset of intestinal adhesion by KN Decoction.

## 5 Conclusion

This study found that Kangnian Decoction inhibited the inflammatory cell response and suppressed fibrous tissue proliferation in rats with intestinal adhesions, thus reducing the degree of abdominal intestinal adhesions. The mechanism underlying these effects may involve the regulation of Glycerophospholipid metabolism, Ether lipid metabolism, Purine metabolism, Arginine biosynthesis, Glycerolipid metabolism, as well as the regulation of the MAPK signaling pathway. These findings may provide new ideas and an experimental basis for establishing treatments and prevention measures for PIAs in clinical practice.

## Data Availability

The data presented in the study are deposited in the ProteomeXchange Consortium repository, accession number PXD055723.
