# Supplementary material for: LC-MS/MS-based metabolomics and proteomics reveal the intervention of Kangnian decoction on the postoperative intestinal adhesion of rats
Source: Front Pharmacol. 2024 Sep 16;15:1382760. doi: 10.3389/fphar.2024.1382760 (PMC11439705; doi:10.3389/fphar.2024.1382760)
Supplement: Supplementary file 1 [file Table1.docx]

Supplementary Material

**Table S1 Summary of differential metabolites**

| **NO.** | **Name** | **Formula** | **RT[min]** | **m/z** | **FC** | | **VIP** |
| --- | --- | --- | --- | --- | --- | --- | --- |
|  |  |  |  |  | **Model/NC** | **KN/Model** |  |
| 1 | 5-Hydroxydihydro-2,3-furandione | C_4_H_4_O_4_ | 0.95 | 175.025 | 0.03 | 6.60 | 1.54 |
| 2 | Vitamin C | C_6_H_8_O_6_ | 0.95 | 175.025 | 0.03 | 6.60 | 1.54 |
| 3 | 7-ketodeoxycholic acid | C_24_H_38_O_5_ | 5.89 | 405.264 | 0.08 | 1.36 | 1.15 |
| 4 | ethyl 3-cyano-2-hydroxy-6-phenylisonicotinate | C_15_H_12_N_2_O_3_ | 1.55 | 267.073 | 0.11 | 1.62 | 1.56 |
| 5 | Purinol | C_5_H_4_N_4_O | 1.25 | 135.032 | 0.14 | 1.72 | 1.60 |
| 6 | L-Glutathione (reduced) | C_10_H_17_N_3_O_6_S | 1.25 | 308.091 | 0.17 | 1.22 | 1.35 |
| 7 | 1-[(8Z,11Z,14Z)-icosatrienoyl]-sn-glycero-3-phosphocholine | C_28_H_52_NO_7_P | 8.29 | 546.355 | 0.18 | 1.71 | 1.42 |
| 8 | Hypoxanthine | C_5_H_4_N_4_O | 0.96 | 137.046 | 0.19 | 1.60 | 1.70 |
| 9 | 1-[(11Z,14Z)]-icosadienoyl-sn-glycero-3-phosphocholine | C_28_H_54_NO_7_P | 8.97 | 548.370 | 0.22 | 1.49 | 1.24 |
| 10 | N-DesmethylZolmitriptan | C_15_H_19_N_3_O_2_ | 5.68 | 274.156 | 0.23 | 1.26 | 1.30 |
| 11 | Allopurinol | C_5_H_4_N_4_O | 1.27 | 137.046 | 0.27 | 1.61 | 1.57 |
| 12 | 1-Linoleoyl glycerol | C_21_H_38_O_4_ | 10.50 | 355.284 | 0.29 | 1.55 | 1.37 |
| 13 | Palmitamide | C_16_H_33_NO | 0.30 | 256.263 | 0.32 | 1.87 | 1.36 |
| 14 | Levulinic acid | C_5_H_8_O_3_ | 0.28 | 117.054 | 0.33 | 1.54 | 1.21 |
| 15 | 1-[(8Z,11Z,14Z,17Z)-icosatetraenoyl]-sn-glycero-3-phosphocholine | C_28_H_50_NO_7_P | 7.71 | 544.339 | 0.33 | 1.36 | 1.46 |
| 16 | 2-oleoyl-sn-glycero-3-phosphocholine | C_26_H_52_NO_7_P | 8.63 | 522.354 | 0.33 | 1.25 | 1.35 |
| 17 | -20-hydroxy-5,8,11,14-Eicosatetraenoic acid | C_20_H_32_O_3_ | 8.43 | 343.224 | 0.36 | 1.21 | 1.39 |
| 18 | Geranylacetone | C_13_H_22_O | 9.00 | 177.164 | 0.36 | 1.68 | 1.42 |
| 19 | 1-arachidonoyl-sn-glycero-3-phosphocholine | C_28_H_50_NO_7_P | 7.87 | 544.339 | 0.38 | 1.57 | 1.36 |
| 20 | 2-Propionylthiazole | C_6_H_7_NOS | 1.36 | 142.032 | 0.38 | 1.46 | 1.58 |
| 21 | Platelet-activating factor | C_26_H_54_NO_7_P | 9.38 | 524.370 | 0.39 | 1.30 | 1.49 |
| 22 | LysoPC(22:4(7Z,10Z,13Z,16Z)) | C_30_H_54_NO_7_P | 8.75 | 572.371 | 0.41 | 1.21 | 1.20 |
| 23 | 1-Oleoyl-rac-glycerol | C_21_H_40_O_4_ | 8.51 | 339.289 | 0.46 | 1.25 | 1.14 |
| 24 | 1-Oleoyl-2-hydroxy-sn-glycero-3-PE | C_23_H_46_NO_7_P | 8.52 | 480.308 | 0.46 | 1.34 | 1.28 |
| 25 | 2-[(11Z,14Z)-icosadienoyl]-sn-glycero-3-phosphoethanolamine | C_25_H_48_NO_7_P | 7.66 | 504.308 | 0.47 | 2.23 | 1.20 |
| 26 | 4-Undecylbenzenesulfonic acid | C_17_H_28_O_3_S | 9.07 | 311.168 | 0.49 | 1.57 | 1.08 |
| 27 | 2-linoleoyl-sn-glycero-3-phosphoethanolamine | C_23_H_44_NO_7_P | 7.78 | 476.277 | 0.51 | 1.57 | 1.01 |
| 28 | L-alpha-Glycerylphosphorylcholine | C_8_H_20_NO_6_P | 0.87 | 258.110 | 0.52 | 1.25 | 1.01 |
| 29 | 6-Methylhept-5-en-2-one | C_8_H_14_O | 4.43 | 127.112 | 0.78 | 1.29 | 1.49 |
| 30 | 4-(2-Carboxy-1-azetidinyl)-N-(3-carboxy-3-hydroxypropyl)threonine | C_12_H_20_N_2_O_8_ | 4.40 | 321.131 | 1.69 | 1.23 | 1.11 |
| 31 | 1-alpha-linolenoyl-2-[(8Z,11Z,14Z)-icosatrienoyl]-sn-glycerol | C_41_H_68_O_5_ | 13.77 | 641.511 | 1.72 | 1.65 | 1.20 |
| 32 | 2-Aminoisobutyric Acid | C_4_H_9_NO_2_ | 0.88 | 102.056 | 1.85 | 0.82 | 1.38 |
| 33 | A-12(13)-EpODE | C_18_H_30_O_3_ | 8.73 | 295.227 | 2.02 | 0.64 | 1.32 |
| 34 | N1,N1-dipropyl-4-(3-azabicyclo[3.2.2]non-3-ylcarbonyl)benzene-1-sulfonamide | C_21_H_32_N_2_O_3_S | 4.68 | 393.224 | 2.03 | 0.80 | 1.38 |
| 35 | D-Glucopyranuronic acid | C_6_H_10_O_7_ | 0.89 | 193.035 | 2.06 | 1.28 | 1.15 |
| 36 | O-ureido-D-serine | C_4_H_9_N_3_O_4_ | 17.14 | 82.537 | 2.15 | 0.78 | 1.12 |
| 37 | 9,10-Dihydroxy-11-(3-pentyl-2-oxiranyl) undecanoic acid | C_18_H_34_O_5_ | 5.96 | 329.233 | 2.17 | 0.75 | 1.45 |
| 38 | 5-AVA | C_5_H_11_NO_2_ | 1.39 | 118.086 | 2.23 | 0.83 | 1.49 |
| 39 | leucoline | C_9_H_7_N | 3.62 | 130.065 | 2.33 | 0.72 | 1.43 |
| 40 | Dihomo-gamma-linolenic acid | C_20_H_34_O_2_ | 12.11 | 307.263 | 2.36 | 0.69 | 1.30 |
| 41 | Adrenic acid | C_22_H_36_O_2_ | 12.24 | 333.278 | 2.40 | 0.76 | 1.48 |
| 42 | aminoproline | C_5_H_10_N_2_O_2_ | 0.75 | 131.081 | 2.56 | 0.80 | 1.35 |
| 43 | Docosatrienoic Acid | C_22_H_38_O_2_ | 13.21 | 333.279 | 2.63 | 0.70 | 1.04 |
| 44 | p-Xylene | C_8_H_10_ | 0.57 | 107.085 | 2.68 | 0.45 | 1.01 |
| 45 | L-Arginine | C_6_H_14_N_4_O_2_ | 0.84 | 175.119 | 2.89 | 0.78 | 1.60 |
| 46 | 4-[(Z)-2-Aminovinyl]phenol | C_8_H_9_NO | 0.95 | 136.075 | 3.04 | 0.73 | 1.32 |
| 47 | 3-Indoleacrylic acid | C_11_H_9_NO_2_ | 3.63 | 188.070 | 3.21 | 0.78 | 1.48 |
| 48 | Sphingosine | C_18_H_37_NO_2_ | 6.88 | 282.279 | 3.28 | 0.75 | 1.59 |
| 49 | 4-Indolecarbaldehyde | C_9_H_7_NO | 3.62 | 146.060 | 3.30 | 0.78 | 1.47 |
| 50 | Guaiacol | C_7_H_8_O_2_ | 2.49 | 188.068 | 3.34 | 0.52 | 1.10 |
| 51 | 5-OxoETE | C_20_H_30_O_3_ | 8.90 | 317.212 | 4.23 | 0.79 | 1.08 |
| 52 | 1-Phenyl-1,3-octadecanedione | C_24_H_38_O_2_ | 12.72 | 359.294 | 4.35 | 0.76 | 1.63 |
| 53 | 2-Methyl-4-quinolinol | C10H9NO | 3.62 | 160.075 | 4.79 | 0.57 | 1.06 |
| 54 | L-Valine | C_5_H_11_NO_2_ | 0.98 | 116.072 | 5.26 | 0.77 | 1.38 |
| 55 | 13,14-dihydro-19(R)-hydroxy Prostaglandin E1 | C_20_H_36_O_6_ | 5.91 | 353.233 | 11.24 | 0.56 | 1.04 |
| 56 | Methyl 14-acetoxy-5-hydroxy-5,9-dimethyltetracyclo[11.2.1.0~1,10~.0~4,9~]hexadecane-14-carboxylate | C_22_H_34_O_5_ | 5.97 | 377.232 | 12.29 | 0.71 | 1.10 |
| 57 | Uric acid | C_5_H_4_N_4_O_3_ | 1.24 | 167.021 | 12.49 | 0.81 | 1.60 |
| 58 | ala-arg | C_9_H_19_N_5_O_3_ | 0.78 | 309.165 | 14.28 | 0.61 | 1.19 |

**Table S2 Differentially expressed proteins**

| **Gene name** | **Accession** | **Fold Change** | | **Gene name** | **Accession** | **Fold Change** | |
| --- | --- | --- | --- | --- | --- | --- | --- |
|  |  | **Model/NC** | **KN/Model** |  |  | **Model/NC** | **KN/Model** |
| Dpep1 | P31430 | 0.62 | 1.34 | Mesd | Q5U2R7 | 1.44 | 0.53 |
| Cfl1 | P45592 | 2.01 | 0.62 | Tax1bp3 | A0A1W2Q5Z6 | 2.23 | 0.63 |
| Pdia5 | Q5I0H9 | 2.72 | 0.14 | H920_07420 | A0A091DGJ8 | 1.75 | 0.54 |
| Ppic | F7FHM1 | 5.44 | 0.33 | Rpl35a | A0A8I6GLI9 | 1.55 | 0.52 |
| Vim | P31000 | 2.04 | 0.38 | Nme1 | Q05982 | 1.60 | 0.69 |
| Snrpd1 | A0A8C6S632 | 1.52 | 0.70 | Col3a1 | P13941 | 1.59 | 0.61 |
| Vcan | A0A0G2K944 | 2.01 | 0.66 | LOC105995859 | A0A1S3G8N9 | 1.58 | 0.67 |
| Tnc | B2LYI9 | 3.91 | 0.42 | Snf8 | A0A1S3GZE8 | 1.94 | 0.38 |
| Rps8 | P62243 | 1.50 | 0.76 | GW7_00978 | G5AU91 | 4.36 | 0.30 |
| Rrbp1 | F1M853 | 1.36 | 0.69 | Pdia6 | A0A0G2JSZ5 | 1.47 | 0.74 |
| Nit2 | A0A8I5ZM02 | 0.50 | 1.78 | Gpx1 | P04041 | 1.94 | 0.55 |
| Rps27a | P62982 | 1.35 | 0.74 | P4hb | P04785 | 1.93 | 0.37 |
| GW7_12661 | G5BEU8 | 0.21 | 1.98 | GW7_05563 | G5AZZ9 | 0.52 | 1.43 |
| Retnlb | Q6DV77 | 0.25 | 1.65 | Spint1 | Q4V8Q2 | 0.54 | 1.66 |
| Aldh9a1 | Q9JLJ3 | 0.27 | 2.91 | Cd9 | A0A8I5ZZU3 | 0.55 | 1.46 |
| Minpp1 | G3V7H2 | 0.30 | 3.45 | Gnao1 | A0A8I5ZT81 | 0.56 | 2.39 |
| Stat5a | Q62771 | 0.33 | 1.48 | Nt5e | A0A8I6AV15 | 0.63 | 1.26 |
| Exoc5 | P97878 | 0.34 | 1.99 | Nid1 | F1LM84 | 0.75 | 1.20 |
| H920_13578 | A0A091D3E2 | 0.35 | 3.43 | Vkorc1 | A0A346T6Q2 | 1.24 | 0.72 |
| Top2a | A0A0G2JUF8 | 0.45 | 1.29 | H920_15478 | A0A091CZ26 | 1.36 | 0.80 |
| NBR_LOCUS16951 | A0A0N4YJ28 | 0.46 | 1.76 | Acyp1 | D4A6X4 | 1.39 | 0.77 |
| Des | Q6P725 | 0.47 | 1.22 | Map1lc3b2 | A0A0G2K9Q7 | 1.43 | 0.74 |
| Pla2g4a | A0A0G2KAA9 | 0.48 | 1.40 | Stx7 | O70257 | 1.43 | 0.66 |
| Nme2 | P19804 | 1.49 | 0.78 | Rpl24 | A0A8C6QV89 | 1.76 | 0.75 |
| Ppib | P24368 | 1.49 | 0.72 | Osbpl10 | A0A8C6QXF3 | 2.05 | 0.64 |
| Srsf2 | Q6PDU1 | 1.52 | 0.68 | Serpinc1 | Q5M7T5 | 2.11 | 0.49 |
| Ranbp1 | D4A2G9 | 1.59 | 0.77 | Rcn1 | D3ZUB0 | 2.15 | 0.80 |
| Erp29 | P52555 | 1.63 | 0.70 | ENSRNOG00000064589 | A0A8I6A4U0 | 2.19 | 0.70 |
| Anxa2 | Q07936 | 1.68 | 0.71 | Fam114a1 | A0A8I6GGB2 | 2.23 | 0.70 |
| LOC120098629 | A0A8I5ZTF4 | 1.75 | 0.68 | Rbm25 | A0A8I6GMR5 | 2.31 | 0.81 |
| Rpl32 | P62912 | 1.75 | 0.72 | Rps29 | P62275 | 2.32 | 0.64 |
| Pabpn1 | A0A1S3FWA9 | 1.75 | 0.48 | Lamtor4 | A0A8I6A858 | 2.35 | 0.68 |
| ENSRNOG00000064207 | A0A8I6A583 | 2.39 | 0.73 | Lrrc25 | A0A8I5ZL91 | 3.03 | 0.40 |
| Serpinh1 | Q5RJR9 | 2.50 | 0.48 | Mt1 | P02803 | 3.84 | 1.38 |
| LOC681544 | A0A0G2K9Y5 | 2.50 | 0.48 | Serpina4 | A0A8I5ZZZ2 | 4.38 | 0.80 |
| Lamp2 | P17046 | 2.52 | 0.61 | Myh10 | A0A8I6A5G4 | 4.45 | 0.61 |
| GW7_15376 | G5BVX3 | 2.54 | 2.73 | Wdr61 | A0A8C6RC95 | 7.90 | 0.53 |
| Ctsa | A0A8I6ARV9 | 2.66 | 0.71 |  |  |  |  |

**Quality control standard of KN Decoction**

**HPLC**

The chromatography procedure was performed using a Agilent high-performance liquid chromatography system. Experimental data were acquired and interpreted exploiting software. The stationary phase was represented by a reverse-phase column (Welch Ultimate XB-C18 column; 250 mm × 4.6 mm, Shanghai, China), while the mobile phase was Acetonitrile as solvent A and Acetonitrile water +0.2% HCOOH as solvent B in 90 min of analysis in total. And the detection wavelength was 254nm.As shown in Table 1, the elution gradient and its timepoints are reported.

| T (min) | Solvent A(%) | Solvent B(%) |
| --- | --- | --- |
| 0-13 | 5-10 | 95-90 |
| 13-40 | 10-20 | 90-80 |
| 40-60 | 20-50 | 80-50 |
| 60-84 | 50-80 | 50-20 |
| 84-90 | 80 | 20 |

**High-performance liquid chromatography fingerprints and the nine main compounds in the KN decoction**

All results indicated that the HPLC analysis method was valid and satisfactory. The HPLC profiles of 15 batches of the KN decoction water extract are shown in Figure S1A. Fifteen coexisting peaks were detected in all analyzed samples; these were labeled 1–19 according to the standard fingerprint of the KN decoction of different grades (Figure S1B ). Indices of similarity among the samples of different grades were in the range of 0.924–0.996 (Table S3). These high degrees of similarity indicated that the compounds found in different grades of the KN decoction were similar. Seven reference compounds were quantitatively analyzed: Chlorogenic acid、Aesculetin、Loganin、Quercetin、Aloe emodin、Rhein acid、Chrysophanol. （Table S4、S5）


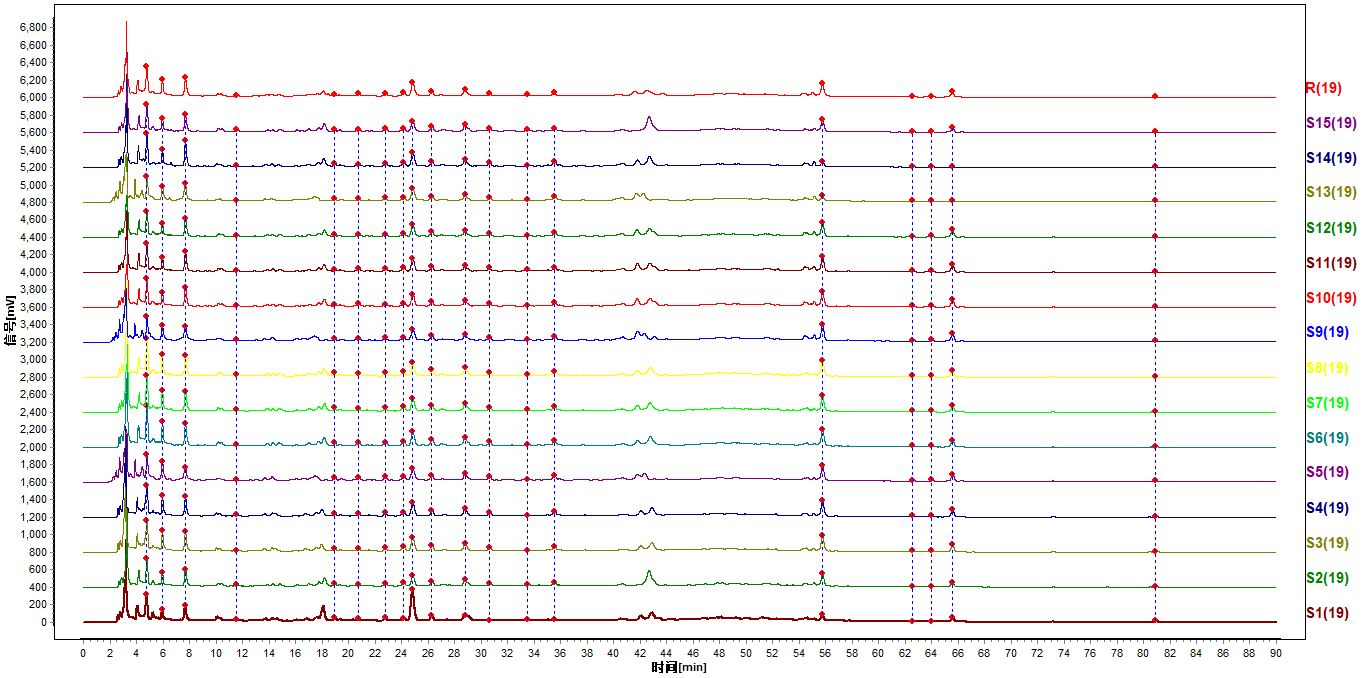
（A）


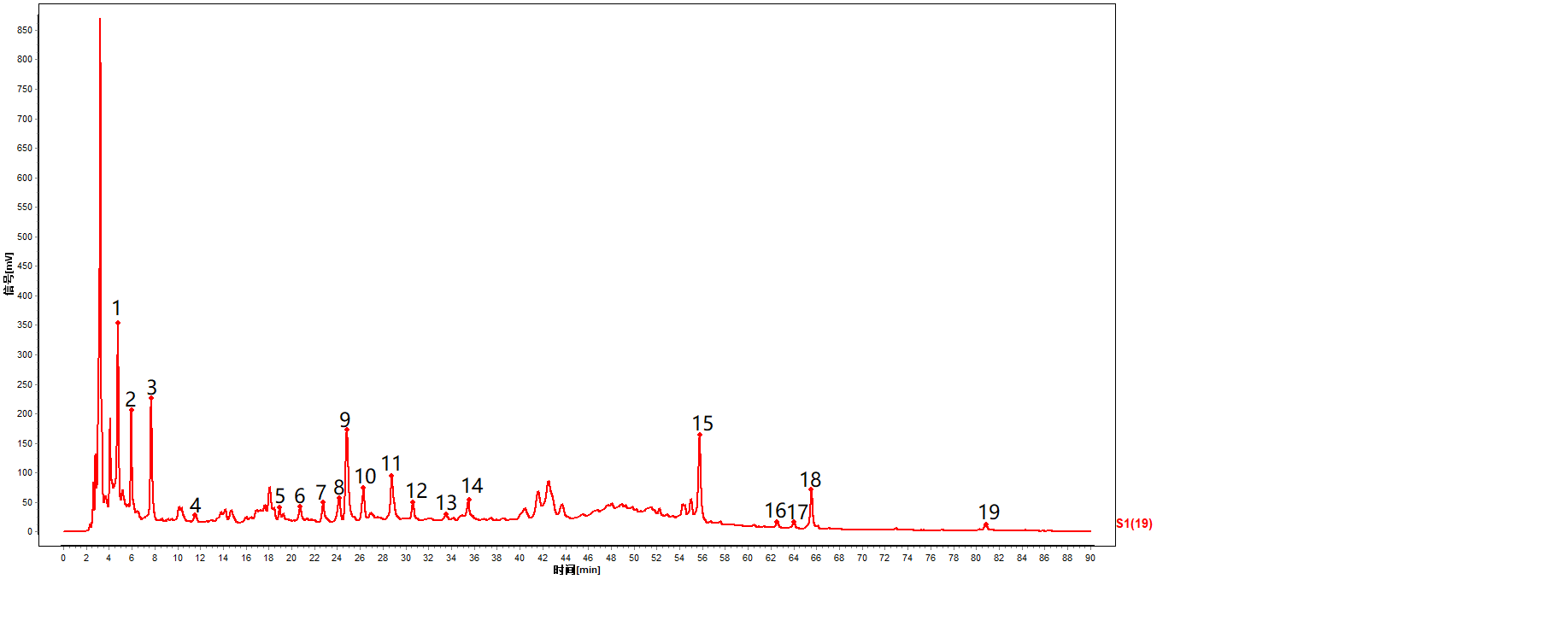
（B）

Figure S1 HPLC fingerprints of samples.((A)Fingerprints of different grades of KN decoction;(B)Standard fingerprints of different grades of KN decoction. .1.unknown;2.unknown;3.unknown;4.unknown;5.unknown;6.unknown;7. Chlorogenic acid;8.unknown;9. Aesculetin;10.unknown;11. Loganin;12.unknown;13.unknown;14.unknown;15. Quercetin;16.unknown;17. Aloe emodin;18. Rhein acid;19.Chrysophanol)

**Table S3 Similarity(Reference to Sample Ten)**

| Sample number | Similarity(  Reference spectrum) | Similarity(  Contrast spectrum) |
| --- | --- | --- |
| 1 | 0.904 | 0.924 |
| 2 | 0.995 | 0.996 |
| 3 | 0.987 | 0.992 |
| 4 | 0.986 | 0.992 |
| 5 | 0.985 | 0.991 |
| 6 | 0.991 | 0.996 |
| 7 | 0.993 | 0.997 |
| 8 | 0.993 | 0.996 |
| 9 | 0.976 | 0.978 |
| 10 | 1.000 | 0.993 |
| 11 | 1.000 | 0.993 |
| 12 | 0.998 | 0.994 |
| 13 | 0.920 | 0.951 |
| 14 | 0.943 | 0.972 |
| 15 | 0.996 | 0.996 |

**Table S4 Linear relationships of various constituents**

| Ingredient | Mathematical equation | Correlation coefficient | Realm（μg/ml） |
| --- | --- | --- | --- |
| Chlorogenic acid | *Y* = 7.2249*X* + 75.729 | 0.9995 | 21.68~346.92 |
| Aesculetin | *Y* = 12.064*X* +68.562 | 0.9999 | 41.65~666.40 |
| Loganin | *Y* = 7.9549*X*+17.588 | 1.0000 | 20.34~325.36 |
| Quercetin | *Y* = 39.071*X*+ 15.5 | 0.9997 | 9.98~159.74 |
| Aloe emodin | *Y* = 32.37*X*+7.3042 | 0.9998 | 1.84~29.40 |
| Rhein acid | *Y* = 34.608*X* – 33.704 | 0.9996 | 5.57~89.18 |
| Chrysophanol | *Y* = 25.184*X*+110.26 | 0.9998 | 2.94~47.04 |

**Table S5 Reasults of content determination of various constituents**

| Batch | Chlorogenic acid（%） | Aesculetin (%) | Loganin（%） | Quercetin（%） | Aloe emodin（%） | Rhein acid（%） | Chrysophanol（%） |
| --- | --- | --- | --- | --- | --- | --- | --- |
| 1 | 0.0132 | 0.256 | 0.125 | 0.0181 | 0.00305 | 0.0171 | 0.00409 |
| 2 | 0.0119 | 0.107 | 0.137 | 0.0537 | 0.00495 | 0.0261 | 0.00425 |
| 3 | 0.0150 | 0.106 | 0.135 | 0.0536 | 0.00515 | 0.0257 | 0.00270 |
| 4 | 0.0135 | 0.108 | 0.123 | 0.0495 | 0.00529 | 0.0254 | 0.00298 |
| 5 | 0.0154 | 0.117 | 0.159 | 0.0581 | 0.00549 | 0.0245 | 0.00312 |
| 6 | 0.0136 | 0.106 | 0.145 | 0.0544 | 0.00525 | 0.0234 | 0.00460 |
| 7 | 0.0145 | 0.112 | 0.150 | 0.0585 | 0.00548 | 0.0242 | 0.00301 |
| 8 | 0.0090 | 0.104 | 0.096 | 0.0486 | 0.00486 | 0.0293 | 0.00292 |
| 9 | 0.0095 | 0.099 | 0.110 | 0.0477 | 0.00529 | 0.0272 | 0.00286 |
| 10 | 0.0100 | 0.100 | 0.110 | 0.0491 | 0.00560 | 0.0276 | 0.00289 |
| 11 | 0.0094 | 0.093 | 0.103 | 0.0459 | 0.00494 | 0.0257 | 0.00288 |
| 12 | 0.0106 | 0.108 | 0.106 | 0.0157 | 0.00159 | 0.0042 | 0.00285 |
| 13 | 0.0137 | 0.110 | 0.121 | 0.0152 | 0.00295 | 0.0044 | 0.00280 |
| 14 | 0.0119 | 0.088 | 0.126 | 0.0414 | 0.00167 | 0.0160 | 0.00362 |
| 15 | 0.0122 | 0.088 | 0.128 | 0.0403 | 0.00173 | 0.0162 | 0.00369 |
| Min | 0.0090 | 0.088 | 0.096 | 0.0433 | 0.00167 | 0.0044 | 0.00270 |
| Max | 0.0154 | 0.256 | 0.159 | 0.0585 | 0.00560 | 0.0293 | 0.00460 |
| Average | 0.0122 | 0.114 | 0.125 | 0.0433 | 0.00422 | 0.0211 | 0.00328 |
